# Long-term upregulation of cortical glutamatergic AMPA receptors in a mouse model of chronic visceral pain

**DOI:** 10.1186/s13041-015-0169-z

**Published:** 2015-11-19

**Authors:** Shui-Bing Liu, Ming-Ming Zhang, Lin-Feng Cheng, Jiao Shi, Jing-Shan Lu, Min Zhuo

**Affiliations:** Center for Neuron and Disease, Frontier Institutes of Science and Technology, Xi’an Jiaotong University, 28 Xianning West Road, Xian, Shaanxi 710049 China; Department of Pharmacology, Pharmacy of School, Fourth Military Medical University, Xian, Shaanxi 710032 China; Department of Physiology, Faculty of Medicine, University of Toronto, 1 King’s College Circle, Toronto, ON M5S 1A8 Canada; Department of Microbiology, Fourth Military Medical University, Xian, Shaanxi 710032 China

**Keywords:** AMPA, Anterior cingulate cortex, Irritable bowel syndrome, AC1

## Abstract

**Background:**

Irritable bowel syndrome (IBS) is one of the most common functional gastrointestinal disorders and it causes long-lasting visceral pain and discomfort. AMPA receptor mediated long-term potentiation (LTP) has been shown to play a critical role in animal models of neuropathic and inflammatory pain. No report is available for central changes in the ACC of mice with chronic visceral pain.

**Results:**

In this study, we used integrative methods to investigate potential central plastic changes in the anterior cingulate cortex (ACC) of a visceral pain mouse model induced by intracolonic injection of zymosan. We found that visceral pain induced an increased expression of AMPA receptors (at the post synapses) in the ACC via an enhanced trafficking of the AMPA receptors to the membrane. Both GluA1 and GluA2/3 subunits were significantly increased. Supporting biochemical changes, excitatory synaptic transmission in the ACC were also significantly enhanced. Microinjection of AMPA receptor inhibitor IEM1460 into the ACC inhibited visceral and spontaneous pain behaviors. Furthermore, we found that the phosphorylation of GluA1 at the Ser845 site was increased, suggesting that GluA1 phosphorylation may contribute to AMPA receptor trafficking. Using genetically knockout mice lacking calcium-calmodulin stimulated adenylyl cyclase subtype 1 (AC1), we found that AMPA receptor phosphorylation and its membrane trafficking induced by zymosan injection were completely blocked.

**Conclusions:**

Our results provide direct evidence for cortical AMPA receptors to contribute to zymosan-induced visceral and spontaneous pain and inhibition of AC1 activity may help to reduce chronic visceral pain.

## Background

Irritable bowel syndrome (IBS) is a common functional gastrointestinal disorder, characterized by colorectal hypersensitivity, abdominal discomfort, bowel dysfunction, and chronic visceral pain [1, 2]. The refractory visceral pain is difficult to treat as the etiology and pathological mechanisms of IBS are not fully understood. Majority of previous studies mainly focused on peripheral modulation and treatment for colorectal hypersensitivity [3, 4]. Recent studies show that enhanced primary sensory afferent is the culprit for pain and colorectal hypersensitivity [2, 5]. In addition, human brain imaging studies revealed that the long-term structural changes in the brain of IBS patients, suggesting the role of central brain areas in IBS related chronic visceral pain [6–9]. Considering the highly plastic nature of cortical synapses, it is expected that enhanced peripheral activity from inflammatory visceral organ could trigger long-term plastic changes in the brain. Potential brain areas involved are the insula cortex and anterior cingulate cortex (ACC) [10, 11]. Our recent studies found that activity-dependent transcription of the Fos gene occurs in the prefrontal cortex, ACC and the insular cortex of the chronic visceral pain animal models [12].

It is well documented that peripheral noxious stimuli trigger a series of neuronal activity along the afferent-ascending somatosensory pathways to central nervous system (CNS). Cumulative evidence supports the notion that forebrain neurons including those in the ACC and insular cortex play a crucial role for pain-related perception [13, 14]. Based on evidence from recent studies, long-term potentiation (LTP) of glutamatergic neurons in the ACC is a key cellular mechanism for pathological chronic pain [14–16]. Postsynaptic recruitment or modification of GluA1-containing α-amino-3-hydroxy-5-methyl-4- isoxazolepropionic acid receptor (AMPA) receptor is observed in the ACC of mice with peripheral nerve injury. Adenylate cyclases (ACs) are enzymes for the regulation and maintenance of different cell function. AC1 is primarily expressed in neurons, and is activated in a calcium-calmodulin (CaM)-dependent manner [17, 18]. AC1 acts as a downstream of glutamate receptors and contributes to chronic pain-related neuronal plasticity in the cortex and spinal cord [19–21].

In the present study, we used integrative methods including biochemical, electrophysiological, pharmacological, and behavioral techniques to explore the role of AMPA receptor in the ACC of mice with intracolonic injection of zymosan. Zymosan treatment causes obvious visceral and spontaneous pain behaviors in mice. Presynaptic neurotransmitter release, postsynaptic responsiveness, and the expressions of GluA1- and GluA2/3-containing AMPA receptor were markedly increased in the ACC of mice injected with zymosan. Pharmacological inhibition of AMPA receptor significantly reduced chronic visceral and spontaneous pain-related behaviors in mice. Genetic deletion of AC1 abolished the enhanced expressions of GluA1 and GluA2/3 in the ACC of mice with chronic visceral pain.

## Results

### Zymosan-induced visceral and spontaneous pain behaviors

**Fig. 1:**
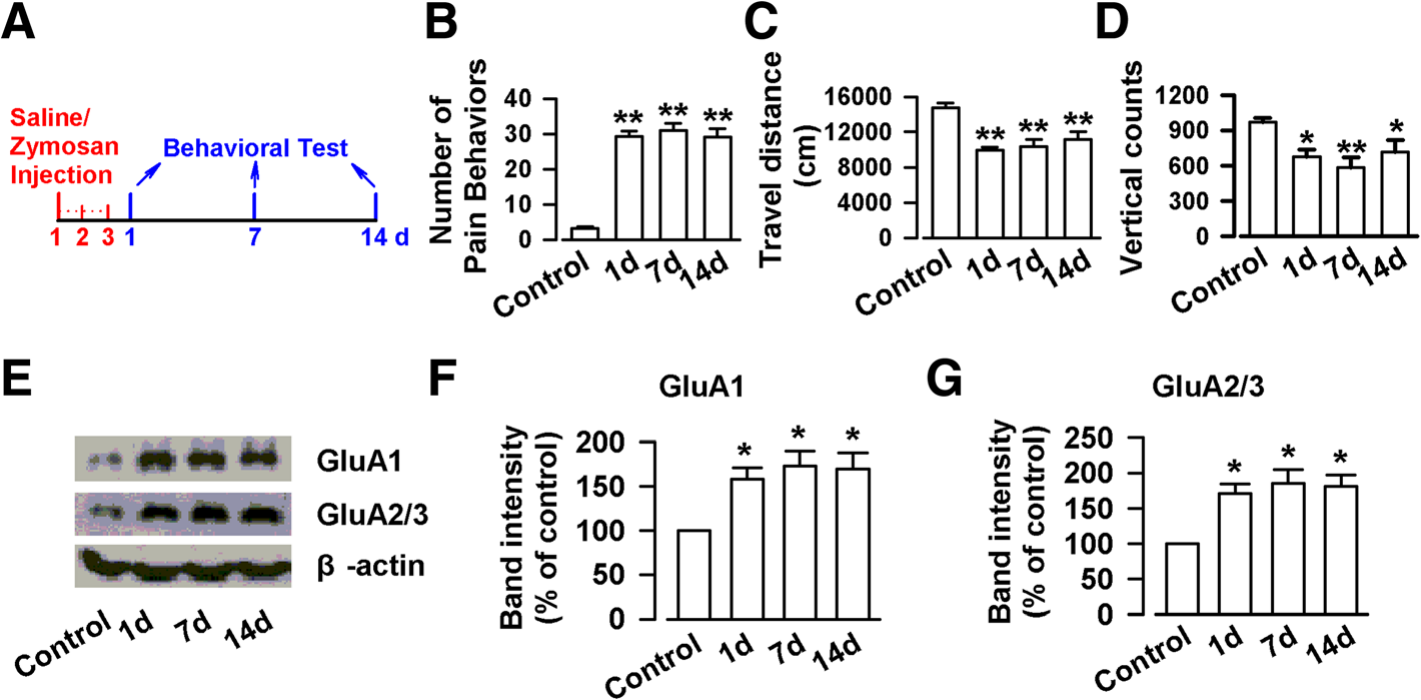
Intracolonic injection with zymosan induced visceral pain-like behaviors and enhanced expression of AMPA receptor in the ACC in mice. **a** The time course for injected with zymosan and behavioral test. **b**-**d** Mice injected with zymosan exhibited obvious visceral pain behavior (**b**), and reduced travel distance (**c**) and vertical counts (**d**) on day 1, 7, and 14 as compared with control. **e** Representative Western blot for GluA1 and GluA2/3 in the ACC obtained from control and zymosan-injected mice. **f**-**g** The expressions of GluA1 (**f**) and GluA2/3 (**g**) were significantly enhanced on day 1, 7, and 14 in the ACC of mice injected with zymosan as compared to those of control mice. N = 6 mice per group. * *P* < 0.05, ** *P* < 0.01 vs. control

Zymosan was used to induce experimental sterile inflammation [22, 23]. Behavioral tests were performed on day 1, 7, and 14 after intracolonic injection with saline or zymosan (Fig. 1a). Our recent study found that adult mice with intracolonic injection of zymosan displayed visceral and spontaneous pain behaviors [12]. Here, we found that intracolonic injection of zymosan obviously induced visceral pain behavior (29.3 ± 1.5 vs. 3.2 ± 0.5, F _(3,20)_ = 60.33, *P* < 0.01, one-way ANOVA, Dunnett T3 test, Fig. 1b), and reduced travel distance (9936.5 ± 326.9 vs. 14774.8 ± 535.6, F _(3,20)_ = 10.44, *P* < 0.01, one-way ANOVA, LSD test, Fig. 1c) and vertical counts (678.8 ± 61.6 vs. 971.2 ± 35.5, F _(3,20)_ = 4.71, *P* < 0.05, one-way ANOVA, LSD test, Fig. 1d) in mice on day 1 after the injection. The pain-like behavior in mice treated with zymosan persisted for two weeks (number of pain behavior: 31.0 ± 2.0, *P* < 0.01 for 7d, 29.2 ± 2.3, *P* < 0.01 for 14d; travel distance: 10359.5 ± 802.8, *P* < 0.01 for 7d, 11557.6 ± 907.2, *P* < 0.01 for 14d; vertical counts: 586.5 ± 85.7, *P* < 0.01 for 7d, 715.7 ± 103.1, *P* < 0.05 for 14d; Fig. 1b, c, and d). There was no significant difference between day 1 and day 7 or day 14 groups in the number of pain behavior (*P* = 0.98 and *P* = 1.0), travel distance (*P* = 0.67 and *P* = 0.22) and vertical counts (*P* = 0.40 and *P* = 0.74), suggesting that zymosan induces long-lasting changes in behaviors.

### Expression of glutamatergic AMPA receptor in the ACC

AMPA receptors mediate most of the basal synaptic transmission in the ACC and contribute to postsynaptic plastic potentiation after peripheral nerve injury [24]. We next wanted to determine if there are alterations in the AMPA receptor subtypes in the ACC of animals with intracolonic injection of zymosan. First, we examined the total expression of GluA1 and GluA2/3 receptors in the ACC, a brain region critical for perception of physical and emotional discomfort [21, 25–27]. We found that the total amount GluA1 and GluA2/3 receptor subtypes were significantly increased in the ACC. On day 1 after intracolonic injection of zymosan, the expressions of GluA1 and GluA2/3 were significantly increased, relative to control mice (GluA1: 158.0 ± 13.0 %, F_(3,20)_ = 6.21, *P* < 0.05, GluA2/3: 171.5 ± 13.3 %, F_(3,20)_ = 8.15, *P* < 0.05, one-way ANOVA, Dunnett T3 test, Fig. 1e, f and g). The increases in the expression levels of GluA1 and GluA2/3 receptors remained high on day 7 (GluA1: 172.8 ± 17.6 %, *P* < 0.05; GluA2/3: 186.0 ± 19.0 %, *P* < 0.05) and day 14 (GluA1: 171.8 ± 16.9 %, *P* < 0.05; GluA2/3: 181.3 ± 16.0 %, *P* < 0.05). These findings indicate that colonic inflammation induced by zymosan injection triggers long-term increase in the expression of AMPA receptors in the ACC.

### Enhancement of AMPA receptor mediated synaptic transmission in the ACC

**Fig. 2:**
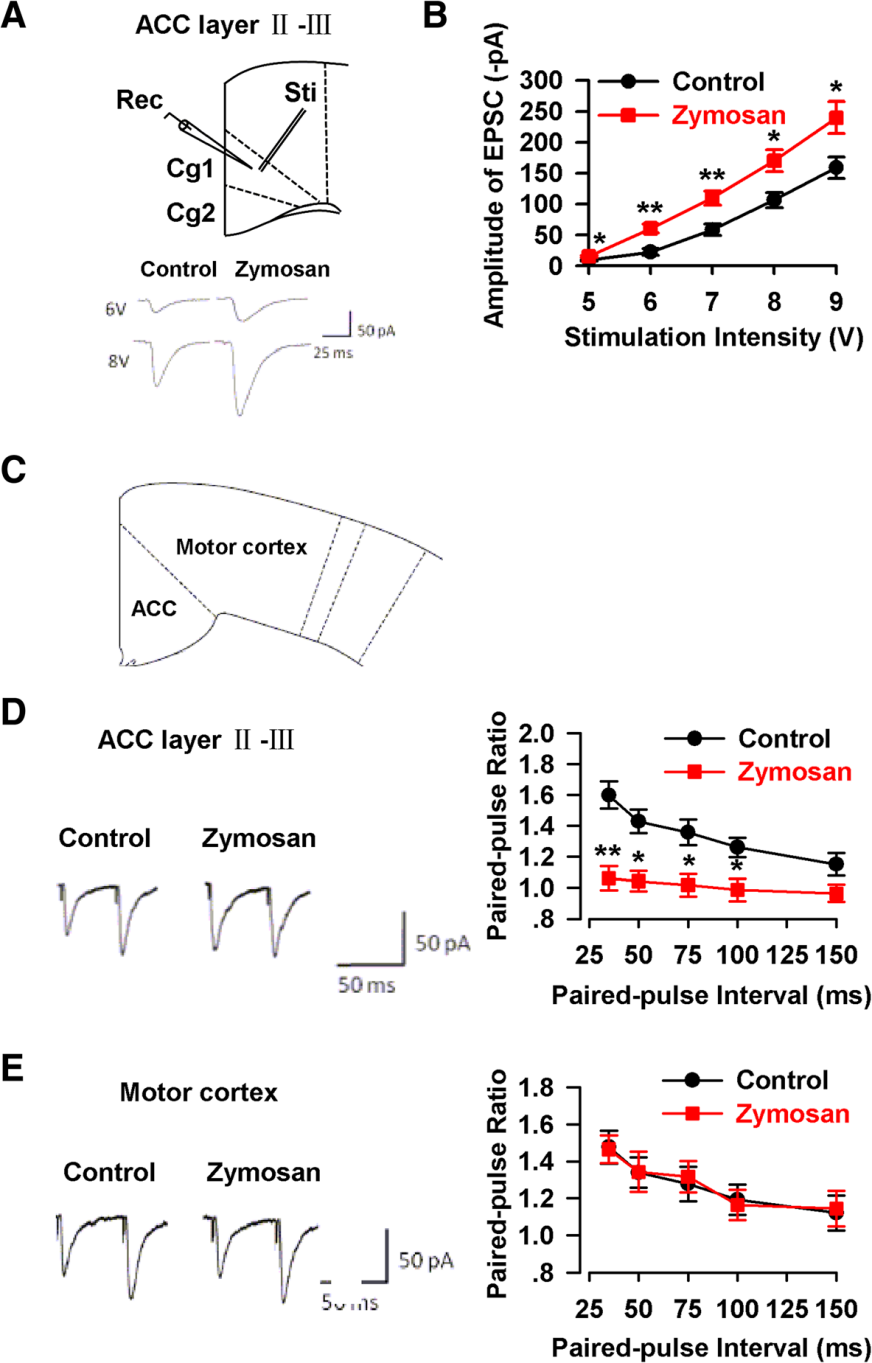
Enhancement of synaptic transmission in the ACC of animal model of IBS. **a** The location of stimulation and recording (top), and representative synaptic input–output curves in the ACC slices from control and zymosan-injected mice (bottom). **b** The amplitude of EPSCs was obviously enhanced in the ACC slice of mice injected with zymosan (*n* = 11/6 mice) as compared with that of control mice (*n* = 10/6 mice). **c** The location of the ACC and motor cortex in coronary slice. **d** Paired-pulse ratio (the ratio of EPSC2/EPSC1) was recorded at intervals of 35, 50, 75, 100, and 150 ms from control (*n* = 9/6 mice) and zymosan-injected mice (*n* = 9/6 mice). PPF was markedly reduced in the ACC of mice injected with zymosan at intervals of 35, 50, 75, and 100 ms. **e** PPF in motor cortex neurons had no difference in control (*n* = 9/6 mice) and zymosan-injected mice (*n* = 11/6 mice). * *P* < 0.05, ** *P* < 0.01 vs. control

To confirm that the upregulation of AMPA receptors may contribute to synaptic transmission in the ACC, we recorded AMPA receptor-mediated EPSCs in pyramidal neurons of mice on day 7 after treated with zymosan (Fig. 2a Top). Pyramidal neurons were identified by the spike frequency adaptation in response to the prolonged depolarizing-current injection. We found that AMPA receptor mediated EPSCs were significantly enhanced in the ACC slices of mice injected with zymosan (Fig. 2a Bottom). Synaptic input (stimulation intensity) – output (EPSC amplitude) curves of AMPA receptor-mediated currents were significantly shifted to the left in the ACC slices of zymosan-treated mice compared to the control mice (5 V: T = −3.24, *P* < 0.05; 6 V: T = −4.29, *P* < 0.01; 7 V: T = −5.68, *P* < 0.01; 8 V: F_(1,50)_ = −2.68, *P* < 0.05; 9 V: T = −2.89, *P* < 0.05; unpaired-*T* test, Fig. 2b). These results indicate that excitatory synaptic transmission is enhanced in the ACC of zymosan-treated mice.

Paired-pulse facilitation (PPF) is a form of short-term synaptic plasticity. To determine if enhanced excitatory synaptic transmission in the ACC is due to presynaptic or postsynaptic mechanisms, we recorded PPF at different stimulus intervals (35, 50, 75, 100, and 150 ms) in the ACC of mice on day 7 after saline or zymosan injection (Fig. 2c). Comparing the recordings from the two groups, PPF was significantly reduced in the ACC of mice after intracolonic zymosan injection (35 ms: T = 4.20, *P* < 0.01; 50 ms: T = 3.44, *P* < 0.05; 75 ms: T = 3.24, *P* < 0.05; 100 ms: T = 2.65, *P* < 0.05; 150 ms: T = 1.61, *P* = 0.17; unpaired-*T* test, Fig. 2d). We also tested the PPF in motor cortex of the same mice and we did not conclude any differences between control and zymosan-treated mice (35 ms: T = 0.11, *P* = 0.92; 50 ms: T = −0.03, *P* = 0.98; 75 ms: T = −0.26, *P* = 0.80; 100 ms: T = 0.22, *P* = 0.83; 150 ms: T = −0.14, *P* = 0.89; unpaired-*T* test, Fig. 2e). These findings suggest that an increase of presynaptic neurotransmitter release may at least in part contribute to the enhanced excitatory synaptic transmission in the layer II-III of the ACC in animal model of chronic visceral pain.

### Enhanced mEPSCs in the ACC

**Fig. 3:**
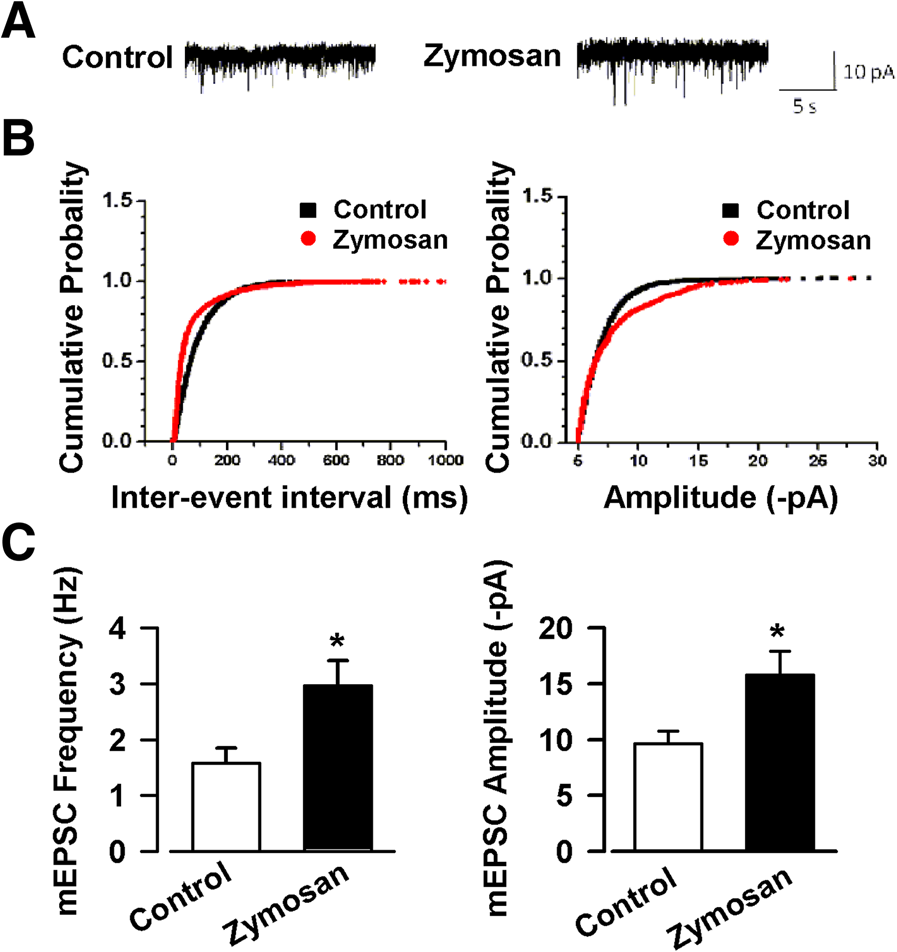
Enhanced mEPSCs in the ACC of animal model of IBS. **a** Representative mEPSCs recorded in pyramidal neurons at a holding potential of −70 mV from control and zymosan-injected mice. **b** Cumulative inter-event interval (left) and amplitude (right) histograms of mEPSCs recorded in slices of control (n = 10/6 mice) and zymosan-injected mice (n = 11/6 mice). **c** Summary plots of mEPSC data. The frequency (left) and amplitude (right) of mEPSCs were significantly enhanced in the ACC slices of mice injected with zymosan. * P < 0.05 vs. control

Next, we tested the miniature excitatory postsynaptic current (mEPSCs), which display the probability of presynaptic neurotransmitter release and postsynaptic responsiveness. The ACC slices of mice on day 7 after saline or zymosan injection were used in the presence of 0.5 μM tetrodotoxin. A robust augmentation of amplitude was observed in the ACC slices from zymosan group (Fig. 3a). Both frequency and amplitude of mEPSCs were significantly increased in the ACC neurons of mice with intracolonic injection of zymosan compared to the control mice (Frequency: T = −3.05, *P* < 0.05; Amplitude: T = −2.82, *P* < 0.05; unpaired-*T* test, Fig. 3b and c). The results suggest that the increases of presynaptic neurotransmitter release and postsynaptic responsiveness both likely contribute the enhanced excitatory synaptic transmission in the ACC of mice with zymosan administration.

### Inhibition of AMPA receptor reduced visceral pain-induced spontaneous pain behaviors

**Fig. 4:**
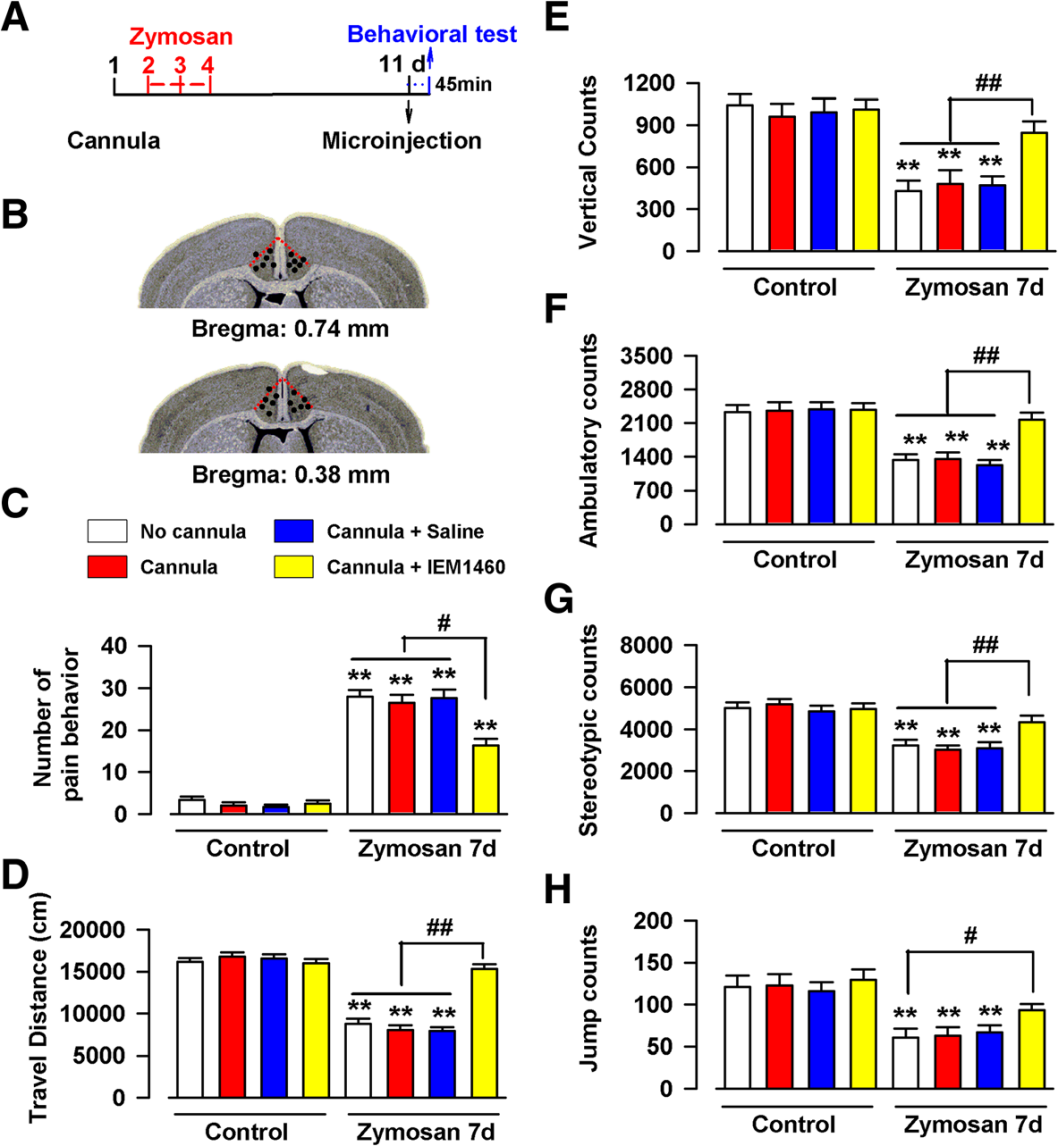
The effect of AMPA inhibitor IEM1460 on behavioral test in the animal model of IBS. **a** The time course of cannulation surgery, zymosan injection, microinjection, and behavioral tests. **b** Schematic showing cannula tip placements in the ACC. C-H) The mice injected with zymosan exhibited obvious visceral pain behavior (**c**), the decreased travel distance (**d**), vertical counts (**e**), ambulatory counts (**f**), stereotypic counts (**g**), and jump counts (**h**) in open field test. However, the microinjection with IEM 1460 totally blocked the effects of zymosan in mice. IEM 1460 had no effect in control mice. *N* = 6 mice per group, ** *P* < 0.01 vs. control (No cannula), ^#^ *P* < 0.05, ^##^ *P* < 0.01 vs. zymosan + IEM 1460

Our biochemical and electrophysiological results consistently suggest that the increased expression of AMPA receptors may contribute to chronic visceral pain. To test this, we performed behavioral experiments in freely moving animals. IEM 1460, a voltage-dependent open-channel blocker of AMPA receptor, blocks GluA2-lacking (Ca^2+^-permeable) receptors (IC_50_ = 2.6 μM) more potently than GluA2-containing receptors (IC_50_ = 1102 μM) [28]. IEM 1460 was microinjected into the ACC bilaterally in mice on day 7 after saline or zymosan injection (Fig. 4a and b), and then behavior tests were started at 45 min after microinjected with IEM1460. Consistent with our previous study, the mice with zymosan treatment exhibited visceral pain behavior (28.0 ± 1.6 vs. 3.3 ± 0.9, F_(7, 40)_ = 78.40, *P* < 0.01; one-way ANOVA, Dunnett T3 test, Fig. 4c), and the decreased travel distance (8758.0 ± 610.8 vs. 16175.5 ± 465.0, F_(7, 40)_ = 61.89, *P* < 0.01; one-way ANOVA, LSD test, Fig. 4d), vertical counts (431.7 ± 73.9 vs. 1042.3 ± 76.9, F_(7, 40)_ = 10.69, *P* < 0.01, one-way ANOVA, LSD test, Fig. 4e), ambulatory counts (1327.3 ± 123.4 vs. 2328.5 ± 147.1, F_(7, 40)_ = 13.89, *P* < 0.01, one-way ANOVA, LSD test, Fig. 4f), stereotypic counts (3244.3 ± 244.8 vs. 5011.3 ± 270.0, F_(7, 40)_ = 12.31, *P* < 0.01, one-way ANOVA, LSD test, Fig. 4g), and jump counts (60.7 ± 10.5 vs. 120.8 ± 13.4, F_(7, 40)_ = 7.06, *P* < 0.01, one-way ANOVA, LSD test, Fig. 4h) in open field test. Microinjection with IEM 1460 markedly inhibited the effects of zymosan on visceral pain behavior (16.3 ± 1.6 vs. 28.0 ± 1.6, *P* < 0.05, Fig. 4c), travel distance (15299.2 ± 556.0 vs. 8758.0 ± 610.8, *P* < 0.01, Fig. 4d), vertical counts (848.2 ± 80.6 vs. 431.7 ± 73.9, *P* < 0.01, Fig. 4e), ambulatory counts (2167.5 ± 150.3 vs. 1327.3 ± 123.4, *P* < 0.01, Fig. 4f), stereotypic counts (4336.0 ± 326.2 vs. 3244.3 ± 244.8, *P* < 0.01, Fig. 4g), and jump counts (93.0 ± 7.8 vs. 60.7 ± 10.5, *P* < 0.01, Fig. 4h). The inhibitory effect of IEM 1460 is relatively selective, and it had no effect on number of pain behavior (*P* = 1.0, Fig. 4c), traveled distance (*P* = 0.82, Fig. 4d), vertical counts (*P* = 0.80, Fig. 4e), ambulatory counts (*P* = 0.80, Fig. 4f), stereotypic counts (*P* = 0.90, Fig. 4g), and jump counts (*P* = 0.58, Fig. 4h) in control mice. These results provide direct evidence that enhanced AMPA receptors contribute to visceral and spontaneous pain-like behaviors in mice with zymosan intracolonic injection.

### Trafficking of AMPA receptors to plasma membrane

**Fig. 5:**
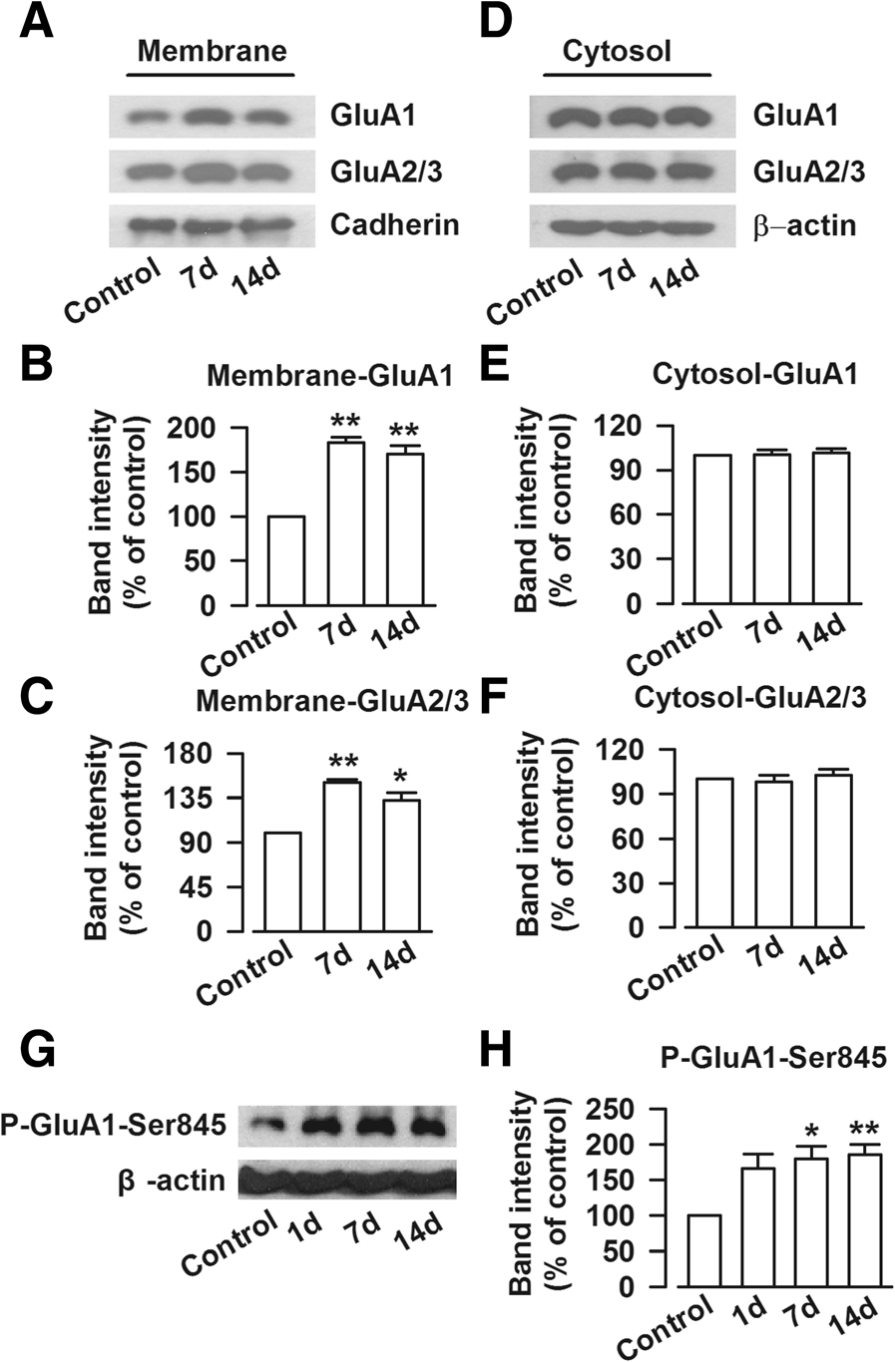
Zymosan injection facilitated the trafficking of AMPA receptor into membrane and phosphorylation of GluA1 at the Ser845 site in the ACC. **a** Representative Western blot for GluA1 and GluA2/3 on membrane protein in the ACC obtained from control and zymosan-injected mice. **b**-**c** The expressions of GluA1 and GluA2/3 on membrane in the ACC were significantly enhanced on day 7, and 14 after mice injected with zymosan. **d** Representative Western blot for GluA1 and GluA2/3 on cytosolic protein in the ACC obtained from control and zymosan-injected mice. **e**-**f**) The expressions of GluA1 and GluA2/3 on cytosol in the ACC had no difference among three groups. **g** Representative Western blot for phosphorylated GluA1 at the Ser845 site in the ACC obtained from control and zymosan-injected mice. **h** The phosphorylation of GluA1 at the Ser845 site was significantly enhanced in the ACC on day 1, 7, and 14 after mice injected with zymosan. N = 6 mice per group, * *P* < 0.05, ** *P* < 0.01 vs. control

**Fig. 6:**
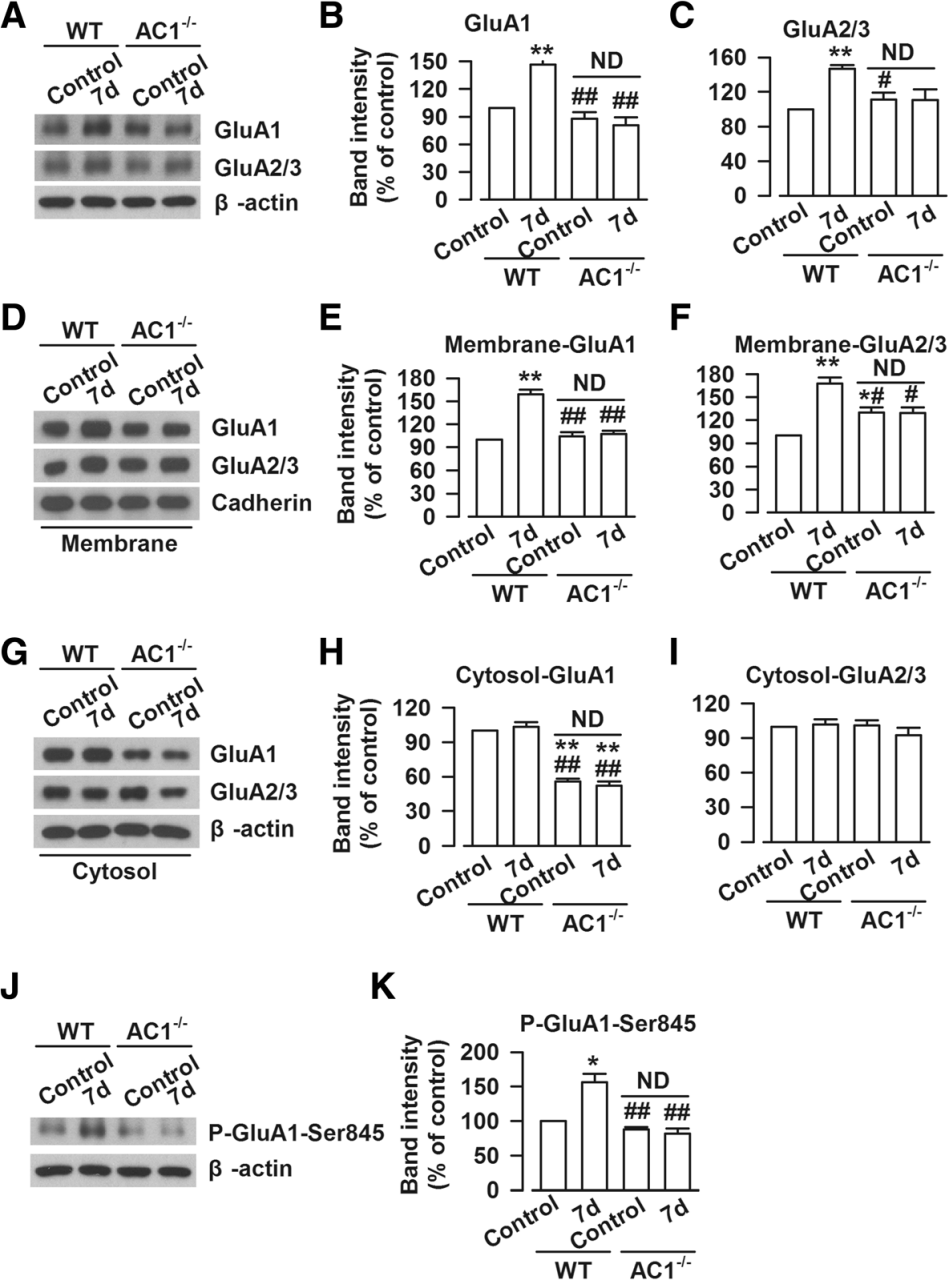
Genetic deletion of AC1 reduced the increased expression of AMPA receptor. **a** Representative Western blot for GluA1 and GluA2/3 in the ACC obtained from WT or AC1^−/−^ control and zymosan-injected mice. **b**-**c** The enhanced expressions of GluA1 (**b**) and GluA2/3 (**c**) were reduced in the ACC of AC1^−/−^ mice on day 7 after zymosan treatment. **d** Representative Western blot for membranous proteins of GluA1 and GluA2/3 in the ACC obtained from WT or AC1^−/−^ control and zymosan-injected mice. **e**-**f** The enhanced membranous expressions of GluA1 (**e**) and GluA2/3 (**f**) were completely or partly blocked in the ACC of AC1^−/−^ mice on day 7 after zymosan treatment. **g** Representative Western blot for cytoplasmic proteins of GluA1 and GluA2/3 in the ACC obtained from WT or AC1^−/−^ control and zymosan-injected mice. **h** The cytoplasmic protein of GluA1 was significantly decreased in the ACC of AC1^−/−^ mice as compared with that in WT mice. **i** There was no difference on cytoplasmic protein of GluA2/3 among four groups. **j** Representative Western blot for phosphorylated GluA1 at the Ser845 site in the ACC obtained from WT or AC1^−/−^ control and zymosan-injected mice. **k** The increased phosphorylation of GluA1 at the Ser845 site was significantly inhibited in the ACC of AC1^−/−^ mice on day 7 after zymosan treatment. N = 6 mice per group, * *P* < 0.05, ** *P* < 0.01 vs. WT control; ^#^ *P* < 0.05, ^##^ P < 0.01 vs. WT for 7d

The results above showed the key role of AMPA receptor in the ACC of mice with chronic visceral pain. Next, we want to characterize the increased AMPA receptors in the ACC of mice after zymosan injection. Previous studies in the hippocampus and ACC show that trafficking of AMPA receptors to the membrane play important roles in synaptic potentiation [29–31]. In our previous study of neuropathic pain, we found that AMPA receptors shift their expression on membranes; and contribute to enhanced sensory transmission in the ACC and insular cortex [20, 32]. To examine possible changes in the distribution of AMPA receptors, we performed biochemical analysis of membrane and cytoplasmic GluA1 and GluA2/3. We selected day 7 and 14 after intracolonic zymosan injection, since the upregulation of AMPA receptors in the ACC reached its peak at these time points. As shown in Fig. 6a, the membrane expression of GluA1 and GluA2/3 were significantly increased on day 7 (GluA1: 182.9 ± 6.4 %, F_(2,15)_ = 45.25, *P* < 0.01; GluA2/3: 150.1 ± 3.0 %, F_(2,15)_ = 32.71, *P* < 0.01, one-way ANOVA, Dunnett T3 test, Fig. 5a, b, and c) as compared with those of control group. On the day 14, the membrane proteins of GluA1 and GluA2/3 sustained high level (GluA1: 169.8 ± 9.5 %, *P* < 0.01; GluA2/3: 132.7 ± 7.1 %, *P* < 0.05, Fig. 5a, b, and c), and there was no difference between day 7 and 14 (GluA1: *P* = 0.60; GluA2/3: *P* = 0.15). Interestingly, the cytoplasmic level of GluA1 and GluA2/3 did not change at both time frames (GluA1: F_(2,15)_ = 0.08, *P* = 1.00 and *P* = 0.94, one-way ANOVA, Dunnett T3 test; GluA2/3: F_(2,15)_ = 0.46, *P* = 0.71 and *P* = 0.58, one-way ANOVA, LSD test, Fig. 5d, e, and f).

### The phosphorylation of GluA1 at the Ser845 site

We next wanted to know which signaling pathway accounts for the increased trafficking of AMPA receptors. In this regard, protein kinase A (PKA) has been shown to play roles in plasticity-related AMPA receptor trafficking [33, 34]. In addition, our previous studies found that AC1-cAMP-PKA activity is critical for the regulation of AMPA receptor phosphorylation and function [19, 32]. Therefore, the phosphorylation of GluA1 at the Ser845 site was detected as phosphorylation of GluA1 at the Ser845 site is a substrate of PKA. Compared with that of control group, the phosphorylation of GluA1 at the Ser845 site was increased on day 7 (179.4 ± 18.2 %, *P* < 0.05) and 14 (185.8 ± 14.4 %, *P* < 0.01) after zymosan injection (F_(3,20)_ = 6.57, one-way ANOVA, Dunnett T3 test, Fig. 5g and h). We also examined phosphorylation of GluA1 at the Ser845 site on day 1 after zymosan injection and we found no statistically significant difference compared to control group (166.4 ± 20.2 %, *P* = 0.10). The percentage increase in the phosphorylation was similar on day 7 and 14, which were comparable with that in the ACC of neuropathic pain animal model [32].

### Calcium-stimulated Adenylyl cyclase 1 (AC1) involved in the visceral pain-induced AMPA receptor changes

Our previous studies reported that AC1 is necessary to induce an increase of AMPA receptor phosphorylation and membrane expression after by nerve injury [32]. Therefore, we want to check if AC1 is required for these changes induced by intracolonic zymosan injection. Indeed, we found that the increased expression of GluA1 and GluA2 receptors were completely abolished or partly reduced in AC1^−/−^ mice (GluA1: 81.0 ± 8.7 % vs. 146.7 ± 4.9 %, F _(3,20)_ = 24.15, *P* < 0.01; GluA2/3: 111.1 ± 11.7 % vs. 146.5 ± 4.6 %, F _(3,20)_ = 7.47, *P* = 0.13; one-way ANOVA, Dunnett T3 test, Fig. 6a, b, and c). The membrane and cytoplasmic proteins of the ACC from AC1 WT and AC1^−/−^ mice were isolated on day 7 after zymosan injection. We found that the increased trafficking of GluA1 to the membrane was completely abolished in the ACC of AC1^−/−^ mice (107.1 ± 4.5 % vs. 159.3 ± 5.7 %, F_(3, 20)_ = 39.09, *P* < 0.01; one-way ANOVA, Dunnett T3 test, Fig. 6d and e). However, the increased trafficking of GluA2/3 was partially reduced in the ACC of AC1^−/−^ mice (129.1 ± 7.6 % vs. 167.8 ± 7.9 %, F_(3, 20)_ = 18.42, *P* < 0.05; one-way ANOVA, Dunnett T3 test, Fig. 6d and f). Furthermore, we noticed that the cytoplasmic expression of GluA1 was reduced in AC1^−/−^ mice as compared with WT mice (52.3 ± 3.6 %, F_(3, 20)_ = 82.95, *P* < 0.01, one-way ANOVA, Dunnett T3 test, Fig. 6g and h). There was no difference on cytoplasmic expression of GluA2/3 between AC1 WT and AC1^−/−^ mice (F_(3, 20)_ = 0.87, *P* = 1.00; one-way ANOVA, Dunnett T3 test, Fig. 6g and i).

We next examined the phosphorylation of GluA1 at Ser845 site. The increased phosphorylation of GluA1 at Ser845 site was completely abolished the ACC of AC1^−/−^ mice (81.3 ± 7.9 % vs. 156.0 ± 12.1 %, F_(3, 20)_ = 21.25, *P* < 0.01; one-way ANOVA, Dunnett T3 test, Fig. 6j and k). This result suggests that the reduced phosphorylation of GluA1 at the Ser845 site may be related to the decreased expression and trafficking of GluA1.

## Discussion

In the present study, we investigated central postsynaptic changes in pain-related cortical area using a mouse model of chronic visceral pain. We showed long-term increases in AMPA receptors and excitatory synaptic transmission in these animals. Using genetic knockout mice, we found that calcium-stimulated AC1 plays an important role in trafficking of GluA1 and GluA2/3 receptors. It may act through phosphorylated GluA1 Ser845. These findings clearly demonstrate that cortical plastic changes of excitatory transmission in the ACC may play important roles in chronic visceral pain and discomfort. These observations also support our recent study that showed the analgesic effects of a selective AC1 inhibitor NB001 [12].

### ACC and chronic visceral pain

Cumulative evidence consistently suggests that neurons of the ACC are involved in pain-related perception, especially in chronic pain [21, 35–38]. Most of these studies about the role of the ACC are limited in animal models using the injuries to somatosensory inputs such as inflammatory pain and neuropathic pain (Table 1); there is less studies of possible synaptic changes in the ACC in animal models using the injuries to visceral organs. Several lines of evidence however indicate that neurons in the ACC may play important roles in visceral pain. In patients with IBS, human imaging data have shown that activity in the brain regions of the ACC, insula and amygdala is enhanced from afferent input by visceral stimulus [39–42]. Similar activation of the ACC, insular cortex, prefrontal cortex and thalamus have been reported in the animal model with visceral pain [43]. In addition, ACC and related supraspinal structures may affect spinal visceral pain transmission. Electrical stimulation of the ACC causes the enhancement of visceromotor response to colorectal distention and ACC lesions reduce visceromotor response to colorectal distention in rats [44]; indicating that similar cortical descending facilitatory modulation may exist for visceral pain [35, 45]. Finally, the GluN2B-containing NMDA receptors, postsynaptic CaMKII, and extracellular signal-regulated kinase-1 and −2 (ERK1/2) in the ACC mediate visceral pain in animals [46–49]. In present study, we provided direct evidence to show the role of AMPA in the ACC in the development of visceral pain via Western blot analysis, electrophysiological record, ACC local drug infusion technique and behavioral tests.

**Table 1:** Studies performed on chronic pain in the ACC of mice

Chronic pain	ACC	References
Inflammatory pain	Increased synaptic GluA1 subunits	Bie et al., 2011
	Activation of mu opioid receptor inhibits the excitatory glutamatergic transmission	Zheng, 2010
	GluA1 but not GluA2 contributes to LTP	Toyoda et al., 2009
	Increased transmitter release and number of available vesicles	Toyoda et al., 2009
	Enhanced presynaptic glutamate release and neuronal cAMP	Wu et al., 2008
	Enhanced presynaptic neurotransmitter release	Zhao et al., 2006
Inflammatory pain & Neuropathic pain	Long-term temporal imprecision of information coding	Li et al., 2014
	Activation of Erk	Wei et al., 2008
Neuropathic pain	Postsynaptic GluA1 accumulation	Chen et al., 2014
	Long-term enhancement of cortical-spinal projecting cells	Chen et al., 2014
	Disinhibition of the ACC	Blom et al., 2014
	Long-term changes in spontaneous membrane-potential oscillations	Ning et al., 2013
	LTD impairment	Kang et al., 2012
	Activation of PKMζ	Li et al., 2010
	Reduced cannabinoid receptor 1 activity	Hoot et al., 2010
	Increased transmitter release	Toyoda et al., 2009
	Presynaptic and postsynaptic amplifications	Xu et al., 2008
Bone cancer pain	LTD impairment	Chiou et al., 2012
Visceral pain	Facilitation of synaptic transmission	Wang et al., 2013
	Increased expression of Fos in the CNS	Zhang et al., 2014
	Long-term enhancement of AMPA receptors in the ACC	This study

### AMPA receptor subtypes and synaptic plasticity in the ACC

AMPA receptors are the major excitatory neurotransmitter receptors and mediate the majority of fast synaptic transmission in central nervous system. There are four subunits of AMPA receptor: GluA1, GluA2, GluA3, and GluA4 [50]. Out of the four subunits, GluA1 is the most significant as it induces the trafficking and integration of AMPA receptors within the synaptic membranes [51]. The vast majority of AMPA receptors in the adult brain contain GluA2, which always render the channel impermeable to calcium. The trafficking of GluA1 is enhanced in the absence of GluA2 [52]. In the hippocampus, increased levels of GluA1 ameliorates memory in mice [53] and synaptic AMPA receptor delivery is essential for learning and memory [54]. Our previous studies have revealed that only GluA1 expression is increased after somatic chronic inflammatory and neuropathic pain and this increased expression of GluA1 may contribute to the related behavioral sensitization [32, 35, 51]. Interestingly, in this study, we found that the expressions of GluA1 and GluA2/3 are both increased in the ACC of animal model. IEM1460 significantly inhibited visceral pain-like behaviors, which suggests that GluA1 is partially involved in visceral pain in the ACC, we could not rule out the role of GluA2/3 in the development of visceral pain. These differences provide a distinct pattern of postsynaptic changes for visceral related chronic pain.

Synaptic plasticity is a crucial mechanism for learning, memory, and chronic pain [14, 15]. At the synaptic level, potentiation of excitatory transmission caused by injury may be mediated by the enhancement of glutamate release from presynaptic terminals and potentiated postsynaptic responses of AMPA receptors [15]. Our electrophysiological data further confirm an enhanced excitatory synaptic transmission in the ACC of zymosan-treated mice, which is attributed to the increases of presynaptic neurotransmitter release and postsynaptic responsiveness. Presynaptic and postsynaptic changes of excitatory transmission are similar to previous researches about somatic inflammatory and neuropathic pain [32, 37]. In addition to the ACC, it is possible that other regions of the brain are also involved in processing of visceral pain, such as the frontal lobe, hippocampus and cornu dorsale [55, 56].

### AC1-cAMP-PKA signaling pathway in the ACC

AC1 catalyzes the conversion of adenosine triphosphate (ATP) to 3’,5’-cyclic AMP (cAMP). AC1-cAMP-PKA signaling pathway is important for somatic inflammation and neuropathic pain. Genetic deletion of AC1 or genetic knock-in lacking phosphorylation of the Ser845 site blocks the enhancement of the GluA1 subunit [20, 35, 57]. AC1 is selectively expressed in neurons and previous studies show that it did not play important roles in learning and memory [17]. The present findings extend the critical role of AC1-cAMP-PKA signaling pathway in chronic visceral pain, and suggest that AC1 selective antagonist NB001 may be used to treat chronic visceral pain [12].

**Fig. 7:**
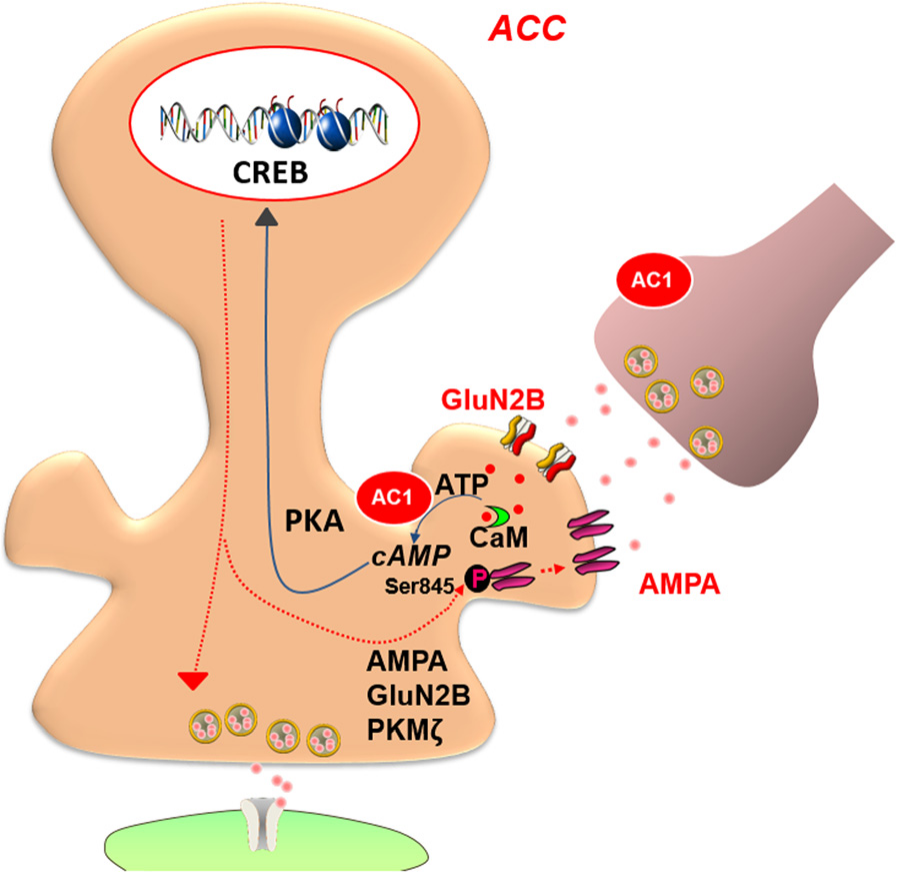
A model showing the molecular mechanism for the upregulation of AMPA receptors in the ACC of IBS animal model. Visceral injuries trigger the release of glutamate in the ACC via ascending visceral pathways. Activation of NMDA receptors lead to calcium fluxes into the postsynaptic membrane. These cause the activation of Ca^2+^/CaM dependent AC1. Activation of AC1 catalyzes the conversion of ATP to cAMP and activates PKA and downstream signaling pathway, which in turn produces the phosphorylation of the GluA1-containing AMPA receptor at Ser845 site, GluN2B and PKMζ. The phosphorylation of the GluA1 at Ser845 site promotes AMPA receptor trafficking to membrane. Increases in postsynaptic AMPA receptors as well as the enhancement of glutamate release significantly enhance excitatory synaptic transmission in the ACC, and then lead to amplification of chronic visceral pain

In Fig. 7, we provided a model for explaining the signaling pathways for chronic visceral pain related plasticity in the ACC. Visceral injuries trigger the release of glutamate in the ACC and subsequent calcium influx into the postsynaptic membrane mediates the activation of Ca^2+^/CaM dependent AC1. Activation of AC1 catalyzes the conversion of ATP to 3’,5’-cyclic AMP (cAMP) and activates PKA. PKA phosphorylates the GluA1-containing AMPA receptor at Ser845 site, which promotes AMPA receptor trafficking to the membrane. In addition, AC1 also contributes to the trafficking of GluA2 and 3 to the membrane. Blocking either AMPA or AC1 could produce powerful antinociceptive effect on spontaneous pain induced by intracolonic injection with zymosan. These results provide novel cortical mechanisms to chronic visceral pain and AC1 inhibitors may be useful for control IBS-related chronic visceral pain in patients.

## Conclusions

In this study, we demonstrate that genetic deletion of AC1 abolishes the upregulation of GluA1 caused by zymosan injection. Together with previous studies of somatic chronic pain models, it strongly indicates the important roles of AMPA GluA1 receptor in cortical sensitization that contribute to chronic somatic and visceral pain. Unlike somatic chronic pain models, we also found that GluA2/3 receptors were upregulated in chronic visceral pain model. The activity of AC1 partially contributes to this upregulation. Future studies are clearly needed to reveal exact molecular mechanism for the upregulation.

## Methods

### Animals

Adult male C57BL/6 mice (age 8–12 weeks) were purchased from Charles River Laboratories (St. Constant, Quebec, Canada) or Animal Center (Xi’an Jiaotong University). AC1 knock out mice (AC1^−/−^, age 8–9 weeks) were bred from a C57BL/6 genetic background. The mice were housed with water and food provided ad libitum under standard laboratory conditions (12 h light/12 h dark, temperature 22-26 °C, humidity 55-60 %). All experiments were performed in accordance with protocols approved by the Animal Care Committee of the University of Toronto and Xi’an Jiaotong University.

### Chronic visceral pain mouse model

To induce visceral pain, mice were treated with intracolonic injection with zymosan (derived from Saccharomyces cerevisiae, Sigma, St. Louis, MO), a Glucan prepared from yeast cell wall characterized as a protein-carbohydrate complex [12]. Briefly, the mice were anesthetized with 1-3 % isoflurane inhalation. A volume of 0.1 ml zymosan suspension (30 mg/ml in saline) was rectally administered into the colons of mice by a 22-gauge, 24-mm-long plastic feeding needle over a period of 2 minutes. Control mice were rectally administered with 0.1 ml saline. Zymosan or saline was given daily for 3 consecutive days.

### Behavioral test

#### Visceral pain behavior

Visceral pain behavior test was performed according to the method of Laird [58]. The numbers of licking abdomen at the absence of other grooming behavior, whole body stretching, flattening the abdomen against the floor, and arched posture for 1–2 sec were recorded in a period of 10 min.

#### Open field test

Open field test was performed from 9:00 am to 12:00 pm in a blind manner on day 1, 7, and 14 after intracolonic injection of zymosan. The mice were acclimatized to the observation room for 30 min before behavioral test. Mice were placed in the center of a novel open field (43.2 × 43.2 × 30.5 cm^3^, Med Associates, St. Albans, Vermont) inside a dimly lit isolation chamber (<50 lux) with a fan. The traval distance, vertical counts, ambulatory counts, stereotypic counts, and jump counts of animal were recorded for 30 min by an activity monitoring system with paired sets of photo beams (Activity Monitor, Med Associates, St. Albans, Vermont).

### Western blot analysis

The ACC of each group of mice were separately dissected in cold ACSF and homogenized in lysis buffer composed of 10 mM tris-Cl (pH 7.4), 150 mM NaCl, 1 mM EDTA, 0.1 % SDS, 1 % Triton X-100, 1 % sodium deoxycholate. The lysis buffer contained a protease inhibitor cocktail and phosphatase inhibitor cocktails 2 and 3 (Sigma, St. Louis, MO). Membrane and cytoplasmic proteins were prepared according to instruction of membrane protein extraction reagent kit (Pierce, Thermo, Rockford, USA). The samples were purified and concentrated according to the instruction provided by SDS-Page Sample Prep Kit (Pierce, Thermo, Rockford, USA). Equal amounts of protein (20 μg) from each sample was separated on a 7.5 % SDS-PAGE gels and transferred to polyvinylidene difluoride (PVDF) membrane to be immunoblotted with anti-GluA1 (dilution ratio, 1:5000, abcam), anti-GluA2/3 (dilution ratio, 1:1000, millipore), anti- phosphorylation-GluA1-Ser845 (dilution ratio, 1:1000, millipore), and β-actin (dilution ratio, 1:50000, Sigma) antibodies at 4 °C overnight. The membranes were incubated with horseradish peroxidase-conjugated secondary antibodies (anti-rabbit/anti-mouse IgG for the primary antibodies) at room temperature for 1 h. The band intensity was expressed relative to β-actin for data quantification.

### In vitro whole-cell patch-clamp recording

Coronal brain slices (300 μm) of the ACC were prepared using standard methods [32, 57, 59]. Slices were transferred to submerged recovery chamber with oxygenated (95 % O _2_ and 5 % CO_2_) artificial cerebrospinal fluid (aCSF) containing (in mM) 124 NaCl, 2.5 KCl, 2 CaCl_2_, 1MgSO_4_, 25 NaHCO_3_, 1 NaH_2_PO_4_, and 10 glucose at room temperature for a minimum of 1 h. Whole-cell patch clamp recordings were performed in a recording chamber on the stage of a BX51W1 microscope equipped with infrared differential interference contrast optics for visualization. Excitatory postsynaptic currents (EPSCs) were recorded from layer II/III neurons with an Axon 200B amplifier (Molecular Devices), and the stimulations were delivered by a bipolar tungsten stimulating electrode placed in layer V/VI of the ACC. AMPA receptor-mediated EPSCs were induced by repetitive stimulations at 0.05 Hz, and the neurons were voltage clamped at −70 mV in the presence of a N-methyl-D-aspartate (NMDA) antagonist AP5 (50 μM). The recording pipettes (3–5 MΩ) were filled with intracellular solution, containing (in mM) 145 K-gluconate, 5 NaCl, 1 MgCl_2_, 0.2 EGTA, 10 HEPES, 2 Mg-ATP, 0.1 Na_3_-GTP, and 10 phosphocreatine disodium (adjusted pH 7.2 with KOH). For miniature EPSC (mEPSC) recording, 0.5 μM TTX was applied to the perfusion solution. Picrotoxin (100 μM) was present throughout the entire recording session for all of the experiments to block GABA_A_ receptor-mediated inhibitory synaptic currents. Access resistance was 15–30 MΩ and monitored throughout the experiment and data were discarded if access resistance deviated >15 % during an experiment. Data were filtered at 1 kHz, and digitized at 10 kHz.

### Cannulation surgery and microinjection

Mice were anesthetized with isoflurane (1–3 %, as needed) inhalation with 30 % oxygen balanced with nitrogen. The scalp of mice were shaved and sterilized with iodine and 75 % alcohol. The head of mice were fixed onto a stereotaxic adapter mounted on a stereotaxic frame (Kopf model 962) and an incision was made on the skull. Two holes of small diameter were drilled on the dura to compensate for each lobes of the ACC located at 0.7 mm anterior to bregma, 0.3 mm lateral to the midline. The guide cannulas were placed into the ACC (1.75 mm ventral to the surface of the skull). After 10 days of recovery time, the mice were restrained in a plastic cone (Braintree Scientific), and a 30 gauge injection cannula was inserted 0.8 mm lower than the guide. Microinjection was performed using a motorized syringe pump (Razel Scientific Instruments) and a Hamilton syringe. N,N,H,-trimethyl-5-[(tricyclo[3.3.1.13,7]dec-1-ylmethyl)amino]-1- pentanaminiumbromide hydrobromide (IEM 1460, Tocris Bioscience) was dissolved in saline was delivered to left and right ACC (1 mM, 0.5 μl per side in 10 min) through the cannula. After the delivery to each side of the brain, the injection cannula was left in place for 2 min to prevent any solution flowing outward. The control mice were microinjected with 0.5 μl saline per side of ACC. Behavioral tests were started at 45 min after injection.

### Data analysis

The Data are presented as the mean ± SEM. Statistical analyses of differences between the two groups were tested by unpaired, two-tailed Student’s *t*-test. Comparison of multiple groups was performed with one-way analysis of variance (ANOVA) (SPSS 19.0). Data were analyzed by one-way ANOVA followed by *post hoc* comparison with least significant difference (LSD) test or Dunnett’s T3 test according to homogeneity test. In all cases, *P* < 0.05 was considered statistically significant.

## Abbreviations

AC1: Adenylyl cyclase subtype 1
ACC: Anterior cingulate cortex
AMPA: α-amino-3-hydroxy-5-methyl-4- isoxazolepropionic acid
ATP: Adenosine triphosphate
Ca^2+^/CaM: Calcium-calmodulin
cAMP: 3’,5’-cyclic AMP
CNS: Central nervous system
ERK1/2: Extracellular signal-regulated kinase-1 and −2
IBS: Irritable bowel syndrome
LTP: Long-term potentiation
mEPSCs: Miniature excitatory postsynaptic currents
NMDA: N-methyl-D-aspartate
PKA: Protein kinase A
